# Wasserstein barycenter regression for estimating the joint dynamics of renewable and fossil fuel energy indices

**DOI:** 10.1007/s10287-023-00436-4

**Published:** 2023-02-04

**Authors:** Maria Elena De Giuli, Alessandro Spelta

**Affiliations:** Department of Economics and Managemen, University of Pavia, San Felice 5, 27100 Pavia, Italy

**Keywords:** Wasserstein barycenters, Density forecast estimation, Renewable energy indices, Fossil fuel indices

## Abstract

In order to characterize non-linear system dynamics and to generate term structures of joint distributions, we propose a flexible and multidimensional approach, which exploits Wasserstein barycentric coordinates for histograms. We apply this methodology to study the relationships between the performance in the European market of the renewable energy sector and that of the fossil fuel energy one. Our methodology allows us to estimate the term structure of conditional joint distributions. This optimal barycentric interpolation can be interpreted as a posterior version of the joint distribution with respect to the prior contained in the past histograms history. Once the underlying dynamics mechanism among the set of variables are obtained as optimal Wasserstein barycentric coordinates, the learned dynamic rules can be used to generate term structures of joint distributions.

## Introduction

The paper proposes a methodology, which provides forecast estimations of the joint density of the market dynamic of fossil-fuel and renewable energy prices, in Europe. We provide a framework for evaluating and improving multivariate density forecast based on optimal transport (OT) theory, which generalizes vector auto-regression modelling for densities. The methodology builds from OT, which aims at finding the less costly way for moving a probability distribution with a given shape to a target probability distribution. In particular, OT algorithms are used to compute the mean of a set of empirical probability measures representing energy prices, under the OT metric. That is, our forecasted densities are Wasserstein barycenter, a measure that minimizes the sum of its Wasserstein distances to each element in a set. Namely, we propose to obtain the estimated evolution of the conditional joint distribution of fossil-fuel and renewable energy prices by directly minimizing the sum of OT distances from the their joint density at time *t* to a set of densities representing the joint dynamics at $t-1,t-2, \ldots ,t-T$.

Against this background, several papers have investigated the interactions between conventional energy resource (oil) prices and renewable ones, showing that a rise in oil prices causes prices of renewable stocks to rise. Henriques and Sadorsky (2008), Kumar et al. (2012) and Managi and Okimoto (2013) have highlighted a significant positive response of clean energy stock prices to oil price shocks due to a substitution effect between traditional fossil fuels and renewable energy. Using multivariate GARCH models, Sadorsky (2012) has analyzed the volatility spillover between oil prices, technology stock prices, and clean energy stock prices, showing that the stock prices of clean energy companies are more highly correlated with technology stock prices than with oil prices in terms of volatility. Wen et al. (2014) have also studied the return and volatility spillover effects between Chinese renewable energy stock prices and fossil fuel stocks, using the asymmetric (BEKK) model, finding that renewable energy and fossil fuel stocks were competing assets, with significant mean and volatility spillovers between them, even though renewable energy stocks were riskier than fossil fuel stocks. In a more recent analysis the spillover approach by Diebold and Yilmaz (2009, 2012, 2014) and its extension proposed by Baruník and Křehlík (2018) is employed to identify the connectedness and its evolution in the electricity market. Using continuous wavelets and non-linear Granger causality in the time-frequency domain, Reboredo et al. (2017) have studied the co-movement and causality between oil and renewable energy stock prices, showing that the dependence was weak in the short run but gradually strengthened towards the long run. In order to study the impact of energy price movements on clean energy stock returns with a multivariate vine-copula dependence setup, Reboredo and Ugolini (2018) have examined dynamics volatility spillovers between clean energy stock prices and different energy prices (oil, gas, electricity and coal) taking into account direct and indirect transmission channel. The connectedness methodology proposed by Baruník and Křehlík (2018) is employed by Ferrer et al. (2018) to investigate the dynamics of return and volatility connectedness over time and across frequencies simultaneously among stock prices of U.S. alternative energy companies, crude oil prices and a number of influential financial variables, namely high technology and conventional energy stock prices, U.S. 10-year Treasury bond yields, the U.S. default spread and the volatility of U.S. equity and Treasury bond markets. Results show that most of return and volatility connectedness is found in the short-term and the crude oil prices are not the key driver of renewable energy companies performance, as opposed to Reboredo and Ugolini (2018). Using a Time-Varying Parameter VAR model with Stochastic Volatility, Urom et al. (2022) have examined the directional predictability from oil price uncertainty to clean energy sectors, characterizing the level of spillovers with wavelet and Cross-Quantilogram techniques. Their analysis reveals that the direction and the magnitude of the response of clean energy sectors to oil market uncertainty vary across sectors, and depend on market conditions and investment horizons while the level of shock spillovers to clean energy sectors from oil price uncertainty is stronger in the intermediate and long-term. By employing a Quantile Vector Autoregression framework, Khalfaoui et al. (2022) have investigated time-frequency transmission and connectedness among green Indices. Evidence from empirical results has shown high spillover and volatility effects among the Indices and a strong connectedness between climate change Indices at extreme lower and upper quantiles.

As mentioned above, the existing prediction methods of co-movement estimation of energy price series have some room for improvement. Indeed, there is a lack of research on the probabilistic prediction of energy price behavior especially for what concern renewable and fossil fuel asset prices. The existing studies mainly focus on the point forecasts, which could not quantify uncertainty and provide less information for policymaking. Moreover, the existing studies have not established a comprehensive factor system based on Europe’s background. To fill up the research gaps discussed above, we propose a flexible and multidimensional approach, which exploits Wasserstein barycentric coordinates for histograms to characterize non-linear system dynamics and to generate term structures of joint distributions (see e.g. Bonneel et al. 2016). Our methodology allows us to estimate the term structure of conditional (in the Wasserstein sense) joint distributions. Wasserstein distances are metrics on probability distributions inspired by the problem of optimal mass transportation. They measure the minimal effort required to reconfigure the probability mass of one distribution in order to recover the other distribution (see Panaretos and Zemel 2019). Initially formulated by Monge (1781) as an intractable non-convex optimization its modern linear programming formulation is due to Kantorovich (1942), and is presented in details in Villani’s monograph (see Villani 2021). We apply this methodology to study the relationships between the market performance of the renewable energy sector and that of the fossil fuel energy sector, in Europe. We focus on the behavior of the European Renewable Index (ERI), which proxies the renewable energy sector, and the MSCI Europe Energy (MSCI EE), which is designed to capture the large and mid cap companies performance across Integrated Oil and Gas segments (see Belhassine 2020). We consider discrete measures supported on the same set of points and we construct the one period-ahead conditional joint distribution as the Wasserstein barycentric interpolation, which minimizes the $\ell _2$ norm between the joint distribution of ERI and MSCI EE at time *t* and the joint distributions realized at $t-1,t-2, \ldots ,t-T$. This optimal barycentric interpolation, obtained as Frechet means in the space of probability measures endowed with the Wasserstein metric (see Agueh and Carlier 2011), can be interpreted as a posterior version of the joint distribution observed at time *t* with respect to the prior contained in the past histograms history. Once the underlying dynamic mechanism among the set of variables are obtained as optimal Wasserstein barycentric coordinates, the learned rules can be used to generate term structures of joint distributions. Using non-parametric methods allows us to remain agnostic about the nature of dynamic interactions between variables of interest, allowing the data to inform us instead. The densities themselves are not assumed to have any specific parametric form, leading to flexible forecasting of future unobserved densities. Moreover, our probabilistic prediction could provide not only the upper and lower of the prediction interval but also the probability density of each point at each time. To the best of our knowledge, this paper is the first document to provide the probability density forecasts for renewable and fossil fuel energy prices. This might become even more relevant in light of the advent of large, exogenous shocks to the financial and socioeconomic fabric of countries such as COVID-19 (Spelta et al. 2021), climate-related natural disasters (Pagnottoni et al. 2022), or human-related disaster such as the Russian–Ukrainian war (Umar et al. 2022), which alter shock propagation mechanisms in financial markets and real economies to different extents (Spelta et al. 2020). Indeed, energy commodity price volatility began mounting in December 2021 when reports of a potential Russian invasion of Ukraine increased and in the first two weeks after the invasion, the prices of oil, and gas went up by around 40%, and 180% respectively.

Our empirical application shows that the one-step ahead predicted joint distribution is approximately Gaussian suggesting market efficiency on the long-run, while on the short-run we observe some departures during crisis phases, where the distribution moves towards negative returns. The two-steps and three-steps ahead forecasts highlight an increasing variance of the model predictions which reflects the higher uncertainty of the forecasts, due to the longer projecting horizon. Moreover, our results reveal the capability of the Wasserstein methodology to describe the joint dynamic of the ERI and MSCI EE indices outperforming benchmark linear models, generally adopted for time series prediction.

The paper proceeds as follows: Sect. 2 contains a brief review of the literature on optimal transport together with the presentation of the technique employed; Sect. 3 provides some descriptive statistics and illustrates the performance of the Wasserstein methodology while Sect. 4 concludes.

## Methodology

### Background on optimal transport and some notation

In order to define our model, we now provide some minimal background on optimal transport and Wasserstein distances, including some relevant notation. Given the simplex $\Sigma _{N} {\mathop {=}\limits ^{ \text{ def. } }}\left\{ h \in {\mathbb {R}}_{+}^{N}; \sum _{i} h_{i}=1\right\}$ of *N* dimensional normalized histograms, and consider a family of *K* reference histograms $\left( h_{1}, \ldots , h_{K}\right)$ in $\Sigma _{N}$. To interpolate between these *K* histograms, we consider barycentric weights $\lambda \in \Sigma _{K}$. For a matrix $A \in {\mathbb {R}}_{+}^{N \times N}$, we write $E(A)=\sum _{i, j} A_{i, j} \log \left( A_{i, j}\right)$ its negative entropy, with the convention $0 \log 0=0$. For two matrices *A*, *B* of the same size, we write $\langle A, B\rangle ={\text {tr}}\left( A^{\top } B\right)$ for their usual inner-product, where $A^{\top }$ is the transpose of *A*. We write $\mathbbm {1}$ for the vector with unit coordinates whose size depends on the context. The $\ell _{\alpha }$ norm for $\alpha \ge 1$ is $\Vert h\Vert _{\alpha }^{\alpha } {\mathop {=}\limits ^{ \text{ def. } }} \sum _{i} h_{i}^{\alpha }$. The Kullback–Leibler divergence between two histograms is $\textrm{KL}(p \mid q) {\mathop {=}\limits ^{ \text{ def. } }} \sum _{i} p_{i} \log \left( p_{i} / q_{i}\right) .$ In this paper, multiplication ( $\prod$ for products of many terms and $\odot$ for two terms) and division / operators between vectors are applied entry-wise, as well as exponential exp and logarithmic log maps.

Of particular relevance to this paper is the recent interest for entropy regularized approaches to solve optimal transport problems (see Cuturi 2013). Instead of a linear programming, entropic smoothing allows the use of Bregman optimization tools (see Bregman 1967), and in particular the Sinkhorn’s algorithm (see Sinkhorn 1964). In the case of entropic regularization, the Wasserstein distance is defined as:

1 $$\begin{aligned} W(p, q) {\mathop {=}\limits ^{ \text{ def. } }} \min _{\Pi \in {\mathbb {R}}_{+}^{N \times N}}\left\{ \langle \Pi , C\rangle +\gamma E(\Pi );\, \Pi {\mathbbm {1}}=p, \Pi ^{\top } {\mathbbm {1}}=q\right\} , \end{aligned}$$

where $\left( p,q\right)$ in $\Sigma _{N}$ are two generic histograms and the matrix *C* quantifies the cost of transporting mass between histogram bins. For instance, if bins are sampled at some locations $x_i$, $x_j$ in a Euclidean space, a common choice for *C* would be $C_{i, j}=\left\| x_{i}-x_{j}\right\| ^{2}$. Indeed, for discrete measures, one can store in the matrix *C* all pairwise costs between points in the supports of the distributions. Usually, the “ground metric” matrix *C* is fixed, representing substitution costs between bins, and shared across several histograms one would like to compare. In the present paper we have employed costs in Euclidean spaces since a nice feature of the Wasserstein distance over an Euclidean space is that one can factor out translations making computations easier. Finally, we assume that the regularization parameter $\gamma$ is positive, which ensures that the optimal solution of this program is unique.

### Wasserstein conditional joint distribution

To produce multi-period conditional distributional forecasts we start by estimating the Wasserstein conditional joint distribution. Consider a time series dataset of $n_{v}$ endogenous variables $v_{i, t}, i=1, \ldots , n_{v}$ and denote by $v_{t}=\left( v_{1, t}, \ldots , v_{n_{v}, t}\right) ^{\prime }$ the vector of date *t* realizations of the *n* variables. Suppose that we have $n_{v^{\star }}$ exogenous predictors $v^{\star }_{t}$, where the predictors are *T* lags of *v*, so that $n_{v^{\star }}=\nu \times n_{v}$ and

$$\begin{aligned} v^{\star }_{t}=\left( v_{t-1}^{\prime }, \ldots , v_{t-\nu }^{\prime }\right) ^{\prime }. \end{aligned}$$

Namely, we are interested in estimating a distributional equivalent to a vector autoregressive model.

We denote $y_t$ the joint distribution of $v_{t}$, found as the *z*-dimensional normalized histogram over the simplex $\Sigma _{Z}$, with $Z= \kappa ^{n}$, being $\kappa$ the number of bins used. We initialize the bins bound as;

2 $$\begin{aligned} \left[ \min \left( v_{j}\right) -\zeta \sigma _{v_{j}}, \max \left( v_{j}\right) +\zeta \sigma _{v_{j}}\right] , \end{aligned}$$

with $\sigma _{v_{j}}$ being the unconditional standard deviation and we discretize the distribution support by employing $\kappa =60$ bins and $\zeta =0.2$. This parameter controls the size of the initial state-space. We employ the $\zeta$ parameter to create a grid point for the bins used for computing the joint distribution.

We define the pairwise cost $C_{i,j}$ with $i,j=1,\ldots ,Z$ as:

$$\begin{aligned} C_{i, j}=\left\| x_{i}-x_{j}\right\| ^{2}, \end{aligned}$$

where $x_i = [{\tilde{d}}_r, {\tilde{d}}_f]$ and $x_j = [{\tilde{d}}_{r'}, {\tilde{d}}_{f'}]$ are two-dimensional returns on the grid described by Equation (2). Thus $C_{i,j}$ describes the cost of moving a mass from a bin indexed by $x_i = [{\tilde{d}}_r, {\tilde{d}}_f]$ to a bin indexed by $x_j = [{\tilde{d}}_{r'}, {\tilde{d}}_{f'}]$.

By starting from the histogram $y_{t}$ representing the joint distribution at time *t*, the dependence structures between variables can be obtained as the weights vector ${\hat{\lambda }}$ which minimizes the $\ell _2$ norm between the projection of $y_{t}$ onto the set of all Wasserstein barycenters $\Omega (\lambda )$ formed by *T* histograms $\left( y_{t-1}, \ldots , y_{t-T}\right)$.

In other words, given the *Z*-dimensional histogram $y_{t} \in$ $\Sigma _{Z}$, the optimal barycentric coordinates of $y_{t}$ within the reference histograms $\left( y_{t-1},y_{t-2}, \ldots ,y_{t-T}\right)$ are computed by finding the vector of probability weights ${\hat{\lambda }} \in \Sigma _{T}$, such that it is an optimal solution to problem:

3 $$\begin{aligned} \underset{\lambda \in \Sigma }{{\text {argmin}}} {\mathcal {G}}(\lambda ) \text { where } {\mathcal {G}}(\lambda ) {\mathop {=}\limits ^{ \text{ def. } }} \frac{1}{2}|| \Omega (\lambda )-y_t||^2_2, \end{aligned}$$

where $\Omega (\lambda )$ defines the Wasserstein barycenters (see Cuturi and Doucet 2014) of the reference *T* histograms $\left( y_{t-1},y_{t-2}, \ldots ,y_{t-T}\right)$ with weights $\lambda$, as:

4 $$\begin{aligned} \Omega : \lambda \mapsto \Omega (\lambda ) {\mathop {=}\limits ^{ \text{ def. } }} \underset{y \in \Sigma _{Z}}{{\text {argmin}}} \sum _{\tau =1}^{T} \lambda _{\tau } W\left( y, y_{t-\tau }\right) . \end{aligned}$$

Although there is no closed-form expression for $\Omega (\lambda )$, Benamou et al. (2015) have shown that the Sinkhorn fixed-point algorithm can be extended to compute Wasserstein barycenters. Following Benamou et al. (2015) we can find the approximate baricenter as:

$$\begin{aligned} \Omega ^{(\ell )}(\lambda ) {\mathop {=}\limits ^{ \text{ def. } }} \prod _{\tau =1}^{T}\left( K^{\top } a_{\tau }^{(\ell )}\right) ^{\lambda _{\tau }} \end{aligned}$$

where

$$\begin{aligned} b_{\tau }^{(\ell +1)} {\mathop {=}\limits ^{ \text{ def. } }} \frac{\Omega ^{(\ell )}(\lambda )}{K^{\top } a_{\tau }^{(\ell )}} \text { and } a_{\tau }^{(\ell +1)} {\mathop {=}\limits ^{ \text{ def. } }} \frac{y_{t}}{K b_{\tau }^{(\ell +1)}} \end{aligned}$$

and $a_{\tau }^{(0)}={\mathbbm {1}}$, $K {\mathop {=}\limits ^{ \text{ def. } }} e^{-C / \gamma }$ is the $Z \times Z$ kernel matrix corresponding to the cost *C* and regularization $\gamma$, such that $\Omega ^{(\ell )}(\lambda ) \underset{\ell \rightarrow \infty }{\longrightarrow }\ \Omega (\lambda )$.

Thus the solution to Equation (3) can be obtained by minimizing a loss function on the approximate barycenter $\Omega ^{(L)}(\lambda )$ computed after a finite number of iterations $L\ge 1$. That is, we solve through quasi-Newton methods the following problem:

5 $$\begin{aligned} \underset{\lambda \in \Sigma }{{\text {argmin}}} {\mathcal {G}}_{L}(\lambda ) \text { where } {\mathcal {G}}_{L}(\lambda ) {\mathop {=}\limits ^{ \text{ def. } }} \frac{1}{2}|| \Omega ^{(L)}(\lambda )-y_t||^2_2. \end{aligned}$$

To determine a stationary point of Equation (5) the gradient of ${\mathcal {G}}_L$ with respect to $\lambda$ can be computed using the chain rule:

$$\begin{aligned} \nabla {\mathcal {G}}_{L}(\lambda )=[\partial \Omega ^{(L)}(\lambda )]^{\top } \nabla {\mathcal {G}}_{L}(\Omega ^{(L)}(\lambda ), y_t). \end{aligned}$$

The gradient of the loss ${\mathcal {G}}_L(\Omega ^{(L)}(\lambda ),y_t)$ evaluated at $\Omega ^{(L)}(\lambda )$ is

$$\begin{aligned} \nabla \frac{1}{2} ||\Omega ^{(L)}(\lambda )-y_t||_{2}^{2}=\Omega ^{(L)}(\lambda )-y_t \end{aligned}$$

Since $\Omega ^{(L)}(\lambda )$ is obtained by recursively applying the same map *L* times, the application of the transposed Jacobian $\left[ \partial \Omega ^{(L)}(\lambda )\right] ^{\top }$ to the vector $\nabla {\mathcal {G}}_{L}(\Omega ^{(L)}(\lambda ), y_t)$ can be computed using backward recursive differentiation (see Neidinger 2010). Finally, following Bonneel et al. (2016), it is possible to prove that:

6 $$\begin{aligned} \nabla {\mathcal {G}}_{L}(\lambda )=\Psi _{\lambda }^{(L)}\nabla {\mathcal {G}}_{L}(\Omega ^{(L)}(\lambda ), y_t)+\sum _{\ell =0}^{L-1} \Phi _{\lambda }^{(\ell )}\left( v^{(\ell )}\right) \end{aligned}$$

where

$$\begin{aligned} \begin{array}{ccc} \Phi _{\lambda }^{(\ell )} {\mathop {=}\limits ^{ \text{ def. } }}\left[ \partial _{\lambda } \Phi \left( b^{(\ell )}(\lambda ), \lambda \right) \right] ^{\top } &{} \text{ and } &{} \Phi _{b}^{(\ell )} {\mathop {=}\limits ^{ \text{ def. } }}\left[ \partial _{b} \Phi \left( b^{(\ell )}, \lambda \right) \right] ^{\top }, \\ \Psi _{\lambda }^{(\ell )} {\mathop {=}\limits ^{ \text{ def. } }}\left[ \partial _{\lambda } \Psi \left( b^{(\ell )}(\lambda ), \lambda \right) \right] ^{\top } &{} \text{ and } &{} \Psi _{b}^{(\ell )} {\mathop {=}\limits ^{ \text{ def. } }}\left[ \partial _{b} \Psi \left( b^{(\ell )}, \lambda \right) \right] ^{\top }, \end{array} \end{aligned}$$

and the vectors $v^{(\ell )}$ are computed using backward recursion, i.e.;

$$\begin{aligned} \forall \ell =L-1, L-2, \ldots , 0, \quad v^{(\ell -1)} {\mathop {=}\limits ^{ \text{ def. } }} \Phi _{b}^{(\ell -1)}\left( v^{(\ell )}\right) \end{aligned}$$

initialized with $$v^{(L)} {\mathop {=}\limits ^{ \text{ def. } }} \Psi _{b}^{(L)}\nabla {\mathcal {G}}_{L}(\Omega ^{(L)}(\lambda ), y_\tau )$$.

For finding ${\hat{\lambda }}$ that minimizes ${\mathcal {G}}_{L}(\lambda )$ we develop an adaptive gradient descent method over a logarithmic domain using the change of variables $\lambda =\frac{e^{\alpha }}{\sum _{\tau } e^{\alpha _{\tau }}} \in \Sigma _{T}$ and carrying out the optimization over $\alpha \in {\mathbb {R}}^{T}$. Following Bonneel et al. (2016) and Malitsky and Mishchenko (2019) we start by setting $\alpha _\tau ^{(0)}=1/T$, and the step size $\mu ^{(0)}=1$ and the step size ratio $\theta ^{(0)}=\infty$. Then, we recursively find the new step size as:

$$\begin{aligned} \mu ^{(k)}={\text {min}}\left\{ \sqrt{1-\theta ^{(k)}}\mu ^{(k-1)},\frac{||\alpha ^{(k)}-\alpha ^{(k-1)}||}{2|| \nabla {\mathcal {G}}_{L}(\alpha ^{(k)}) -\nabla {\mathcal {G}}_{L}(\alpha ^{(k-1)})||} \right\} \end{aligned}$$

and we update $\alpha$ and $\theta$ as:

$$\begin{aligned} \alpha ^{(k)}= & {} \alpha ^{(k)}-\mu ^{(k)}\nabla {\mathcal {G}}_{L}(\alpha ^{(k)}) \\ \theta ^{(k)}= & {} \frac{\alpha ^{(k)}}{\alpha ^{(k-1)}}. \end{aligned}$$

### Model selection

The number *T* of lagged histograms for the derivation of the barycentric interpolation is determined by exploiting the Wasserstein distance as an error measure for comparing different probability distributions. In this section we make explicit the dependence of $\Omega ^{(L)}(\lambda )$ from the number of lagged histograms $\left( y_{t-1},y_{t-2}, \ldots ,y_{t-T}\right)$ used to solve our optimization problem. Then, we derive a criterion based on the minimization of the $\ell _2$ Wasserstein distance between $y_{t}$ and $\Omega ^{(L)}_T(\lambda )$ for different values of *T*.

In formulae we choose the optimal lag $T^{\star }$ as:

7 $$\begin{aligned} T^{\star } \in \min _{T} W_{2}\left( \Omega ^{(L)}_T(\lambda ), y_t\right) ^{2}, \end{aligned}$$

where $W_{2}\left( \Omega ^{(L)}_T(\lambda ), y_t\right) ^{2} = \sum _{i, j} \Xi _{i,j}^{\star } C_{i, j}$ and where and $\Xi ^{\star }$ is the optimal transport map defined as:

8 $$\begin{aligned} \Xi ^{\star } \in \min _{\Xi \in {\mathbb {R}}_{+}^{Z \times Z}}\left\{ \langle \Xi , C\rangle +\gamma E(\Xi ); \Xi {\mathbbm {1}}=\Omega ^{(L)}_T(\lambda ), \Xi ^{\top } {\mathbbm {1}}=y_t\right\} . \end{aligned}$$

### Joint distribution forecast

In order to predict joint probabilities, we exploit the learned dependence structure between densities and we apply the estimated model to the realized value at time *t* to determine its value in the next time step $t+1$. We keep fixed the optimal Wasserstein baricenters ${\hat{\lambda }}$, which minimizes the $\ell _2$-norm between the joint probability distribution at time *t* and the joint distributions at $t-1,t-2, \ldots t-T$, and we compute the predicted *Z*-dimensional histogram at $t+1$, as the Wasserstein baricenter among the joint distribution realized at $t,t-1, \ldots t-T-1$. In formulae:

9 $$\begin{aligned} y_{t+1}({\hat{\lambda }}) {\mathop {=}\limits ^{ \text{ def. } }} \underset{y \in \Sigma _{Z}}{{\text {argmin}}} \sum _{\tau =1}^{T} {\hat{\lambda }}_{\tau } W\left( y, y_{t-\tau -1}\right) . \end{aligned}$$

We then proceed by substitution to obtain multiple-step ahead forecasts. Namely, by keeping fix ${\hat{\lambda }}$, to forecast the joint density at time $t+2$, we use the information set $I^{t+1}=\{ y_{t+1}({\hat{\lambda }}), y_{t}, \ldots , y_{t-T-2} \}$, while for three-step ahead $I^{t+2}=\{ y_{t+2}({\hat{\lambda }}),y_{t+1}({\hat{\lambda }}), y_{t}, \ldots , y_{t-T-3} \}$.

## Results

### Preliminary descriptive statistics

To study the relationships between the market performance of the renewable energy sector and that of the fossil fuel, we focus on the behavior of the European Renewable Index (ERI) which proxies the renewable energy sector and the MSCI Europe Energy (MSCI EE) which is designed to capture the large and mid cap companies performance across Integrated Oil and Gas segments in Europe.

**Table 1:** Yearly summary statistics

Year	Mean		Min		Max		Std		Skew		Kurt	
	*ERI*	*MSCI EE*	*ERI*	*MSCI EE*	*ERI*	*MSCI EE*	*ERI*	*MSCI EE*	*ERI*	*MSCI EE*	*ERI*	*MSCI EE*
2003	0.0170	0.0536	− 0.121	0.0132	0.114	0.1319	0.1227	0.0678	− 0.5451	0.7061	1.50	1.50
2004	0.0159	0.0188	− 0.0721	− 0.0217	0.1173	0.0717	0.0563	0.0296	0.2444	0.3089	2.3392	2.1004
2005	0.0444	0.0194	− 0.1113	− 0.0989	0.1683	0.130	0.0861	0.0596	− 0.2783	− 0.1986	2.1055	2.9781
2006	0.0443	0.0122	− 0.1164	− 0.0889	0.2595	0.125	0.0933	0.0589	0.6911	0.109	3.825	2.4643
2007	0.0419	0.0202	− 0.0248	− 0.0523	0.1106	0.0767	0.0459	0.0420	− 0.1302	− 0.344	1.7348	1.6789
2008	− 0.0771	− 0.0421	− 0.4222	− 0.1921	0.0867	0.1153	0.1691	0.0977	− 1.019	− 0.0962	2.5957	1.9313
2009	− 0.00390	0.0178	− 0.1405	− 0.0989	0.2244	0.1283	0.0965	0.0591	0.7527	− 0.2617	3.9208	3.0644
2010	− 0.0383	0.00740	− 0.1713	− 0.1347	0.1152	0.0964	0.0913	0.0732	0.1297	− 0.4258	1.819	2.2311
2011	− 0.0387	− 0.00160	− 0.2069	− 0.133	0.1448	0.1575	0.0853	0.0768	0.2791	0.2087	3.734	3.1078
2012	− 0.0220	− 0.000500	− 0.0969	− 0.1328	0.0780	0.0540	0.0640	0.0512	0.3853	− 1.4334	1.6758	4.7691
2013	0.0533	0.0119	− 0.0372	− 0.0361	0.1732	0.0599	0.0761	0.0293	0.2684	0.1275	1.7344	2.0794
2014	0.00680	− 0.0123	− 0.0826	− 0.0948	0.1315	0.0556	0.0660	0.0531	0.5548	− 0.1275	2.1982	1.651
2015	0.0326	− 0.0240	− 0.0709	− 0.1022	0.1325	0.1044	0.0589	0.0661	− 0.0314	0.9261	2.2427	2.4931
2016	− 0.00240	0.0171	− 0.0877	− 0.0298	0.0359	0.0820	0.0378	0.0409	− 1.0338	0.3571	3.1084	1.7622
2017	0.00580	0.00170	− 0.1276	− 0.0396	0.0631	0.0833	0.0511	0.0364	− 1.5436	0.9821	4.9019	3.0566
2018	0.00730	− 0.0168	− 0.1126	− 0.1067	0.1295	0.0897	0.0649	0.0621	− 0.0817	− 0.2141	2.7651	2.0802
2019	0.0292	0.00620	− 0.0368	− 0.0929	0.1524	0.0981	0.0494	0.0563	1.188	− 0.3243	4.3071	2.4633
2020	0.0543	− 0.0351	− 0.1405	− 0.3516	0.2048	0.2482	0.0899	0.1527	− 0.5924	− 0.1559	3.2271	3.2998
2021	− 0.0180	0.0251	− 0.1146	− 0.0769	0.0807	0.1399	0.0618	0.0611	0.2999	− 0.000900	2.1076	2.6599
2022	0.00470	0.0668	− 0.1178	0.00890	0.1521	0.1424	0.1122	0.0566	0.3644	0.4788	1.9568	1.9071

Summary statistics of the reference indices returns. ERI identifies the European Renewable Index while the tag MSCI EE refers to the MSCI Europe Energy index. For each asset we report the yearly average (Mean) value of the quantity along with minimum (Min), the maximum (Max), the standard deviation (Std), the skewness (Skew), and the kurtosis (Kurt)

Table 1 shows summary statistics of the main quantities used throughout the paper, namely the monthly returns of the ERI and MSCI EE indices. The measures are yearly averages of the values. In the table we report the average value, the minimum and the maximum of each quantity along with the standard deviation, the skewness and the kurtosis of the distribution. Notice how, during the phase 2008–2012, the ERI index suffered higher losses compared with the MSCI EE, displaying also an increasing standard deviations. In the last part of the sample, the MSCI EE index reports higher returns due to the increasing energy prices related to the fear and the outbreak of the Ukrainian war.

**Fig. 1:**
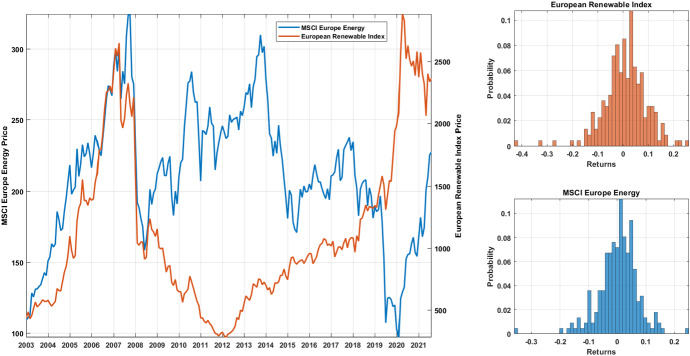
Price dynamics and return distributions. The figure reports the price evolution of the European Renewable Index and the MSCI Europe Energy index (left panel) together with the returns distributions (right panels). The blue color identifies the MSCI Europe Energy index while the European Renewable Index is depicted in orange (color figure online)

Figure 1 supports the finding provided in Table 1 by showing the price evolution of the two indices in the main panel and their pooled returns distributions in the right panels. From the figure, two market trends are clearly visible. Up to the 2008–2009 financial crisis, the two series have correlated behaviors, but after that period, the indices start moving in opposite direction, with an increasing ERI index price and a falling performance of the MSCI EE up to the year 2020. This suggests a change in the investor behavior which find the renewable energy sector more attractive then the fossil fuel. The last part of the sample highlights an inversion of this trend. Looking at the return distributions we observe heavy negative tails in both the two indices, with a more pronounced one in the renewable energy sector.

**Fig. 2:**
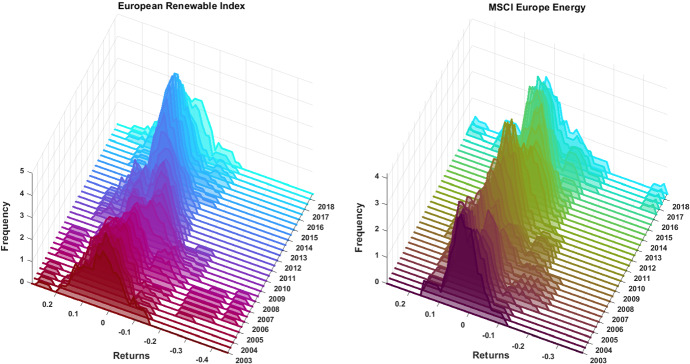
Marginal distributions evolution. The figure shows the dynamics of the marginal distributions of European Renewable Index (left panel) and the MSCI Europe Energy index returns (right panel). Histograms are obtained by employing a rolling window of 3 years

To have a deeper understanding on the evolution of the returns of the renewable and fossil fuel indices, we show in Fig. 2 the dynamic of the marginal distributions of the ERI (left panel) and of the MSCI EE (right panel). These distributions are obtained with a rolling window of three years and emphasized the higher volatility induced by the Global Financial Crisis on the ERI returns and the effect of Ukrainian war on the MSCI Europe Energy at the end of the sample.

### Evolution of the joint distribution

**Fig. 3:**
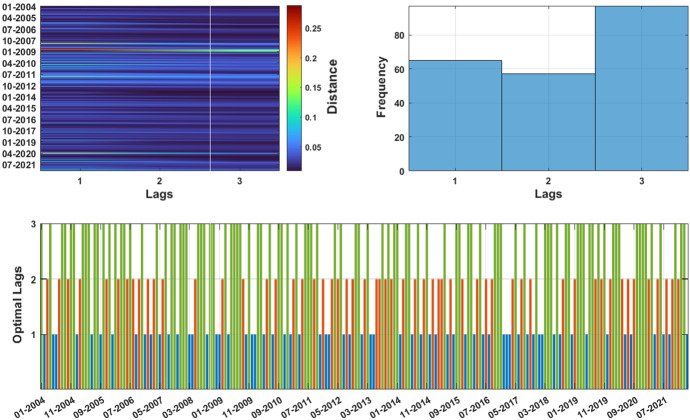
Model selection. The figure reports the $\ell _2$ Wasserstein distance between the estimated model and the joint distribution of assets returns (upper-left panel), together with the optimal lag distribution (upper-right panel) and the evolution of the optimal lag through time (lower panel). The optimal lag represents the information set with the minimum Wasserstein distance

We begin by illustrating the results related to the optimal lag selection. Figure 3 reports, on the left panel, the monthly Wasserstein distance between the empirical joint distribution and the estimated model for different lags. The bottom panel identifies the optimal lag for each month with colored bars, while the right panel shows the distribution of the optimal lags. On average, the model selection procedure suggests the inclusion of the highest number of predictors in the information set adopted for the model estimation. On the other hand, when the univariate distributions present long tails (as in period 2006–2007 for the European Renewable Index and in 2017–2018 for the MSCI Europe Energy index) the selection scheme highlights the reduced number of optimal lags selected.

**Fig. 4:**
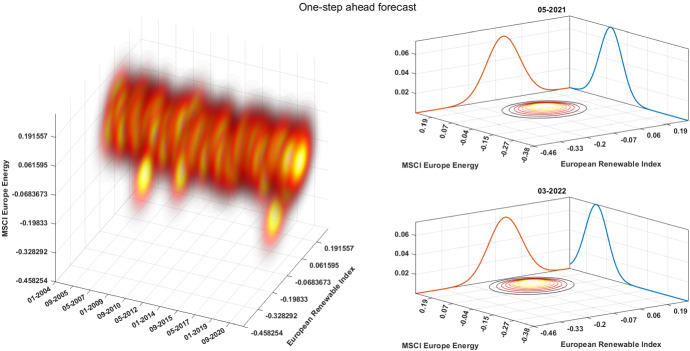
One-step ahead forecast. The figure shows the one-step ahead conditional joint distribution between asset returns. The left panel reports the evolution of the predicted joint distribution as contour plots, with darker shades of red corresponding to lower probability densities. The right panels display the joint distribution in specific time frames along with the marginals of the two indices (color figure online)

Figure 4 plots the estimated joint distribution of the renewable and fossil fuel indices returns at one month ahead over time. Contour plots shaped like symmetric disks corresponding to (joint) Gaussian distributions approximately centered around zero. This means that the predictied distribution suggests market efficiency on the long-run, being equally uncertain about both improvements and deteriorations to both energy indices. On the other hand, we observe some crisis phases, in which the joint distribution moves towards negative returns. The right panels highlight this feature by reporting a business as usual joint distribution (upper panel) and a crisis phase joint distribution (lower panel), together with the marginals for both the MSCI European Energy index and European Renewable Index. Interestingly, the lower panel shows the effect of the Ukraine war on the energy indices. While the European Renewable Index is not affected, the MSCI Europe Energy displays an increase of the returns due to the war fear.

**Fig. 5:**
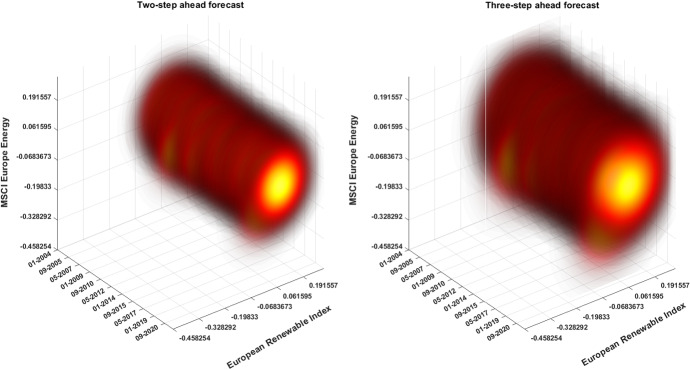
Joint distributions across horizons. The figure shows the two-step (left panel) and the three-step (right panel) ahead conditional joint distribution of asset returns. The evolution of the predicted joint distributions are reported as contour plots, with darker shades of red corresponding to lower probability densities (color figure online)

Figure 5 reports the two-step (left panel) and three-step ahead (right panel) forecasts of the joint distribution. The figure shows that the joint distribution of renewable and fossil fuel energy indices returns exhibits distinctly Gaussian behavior during periods under analysis, with a slightly decrease of returns forecasts during market crisis phases. Moreover the larger contours highlight the increasing variance of the model prediction which reflects the higher uncertainties of the forecasts due to the longer projecting horizon.

**Fig. 6:**
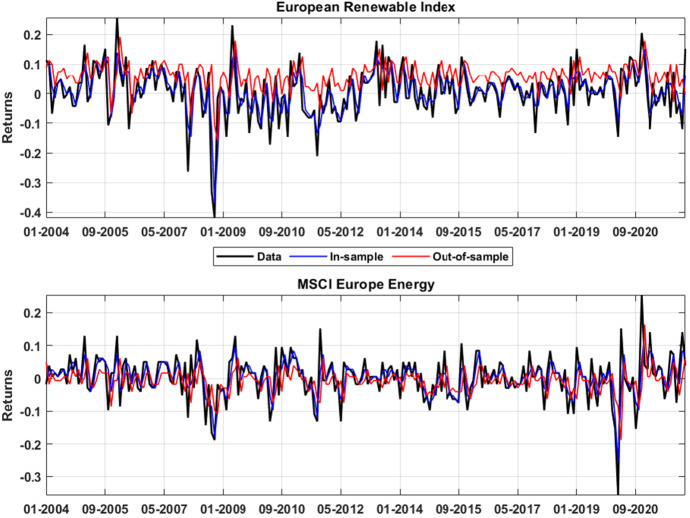
Asset returns one-step ahead point estimate. The figure reports the point estimates of the indices’ returns. The upper panel shows the returns dynamic for the European Renewable Index while the lower panel displays the same quantities for the MSCI European index. The black line identifies the actual returns while the blue line reports the in-sample dynamic. The red lines shows the predicted one-step ahead point estimates for assets returns

Figure 6 shows the in-sample and one-month ahead out-of-sample point estimates for the European Renewable Index (upper panel) and for the MSCI Europe Energy index (lower panel). Point estimates are obtained by picking the indices returns which support the highest probability of the marginals. Figure 6 shows that the in-sample estimate, described by the blue line, is virtually indistinguishable from the real data (black line), for both the renewable an fossil fuel energy indices thus suggesting the model ability to explain the returns dynamic. On the other hand, the point forecasts depicted in red, deviate from the data, especially for the European Renewable Index, but, on average, they are able to correctly detect the returns behavior.

We now turn to evaluating the out-of-sample performance of our model relative to a standard benchmark: a VAR model with Gaussian errors. The comparison of the point estimate predictions derived from our model with those generated by the linear VAR model allows us to evaluate whether Wasserstein barycenters of statistical distributions produce superior forecast performances with respect to time-series linear models. For the latter model, we employ the Bayesian Information Criterion (BIC) to select the optimal lags and we exploit the Root Mean Squared Error (RMSE), which measures the differences between values predicted by the models and the values observed, as a prediction accuracy measure.

**Fig. 7:**
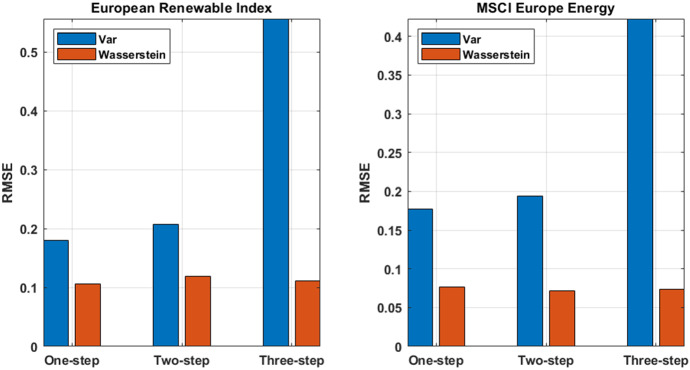
Prediction accuracy measures. The figure shows the RMSE related to the point estimate forecast produced by the proposed methodology against VAR model predictions. The left panel shows, for one, two and three-step ahead forecasts, the error measure for the European Renewable Index while the right panel displays the same quantities for the MSCI European index

Figure 7 displays the goodness of the predictions through the RMSE for one, two and three-step ahead returns forecasts for the European Renewable Index (left panel) and for the MSCI European Energy index (right panel). The forecasting procedure adopting the Wasserstein distance generates the best performance in all the cases, with the MSCI European Energy Index return forecasts that yields the lowest RMSE values. Moreover, while the error produced by the VAR model increase as long as longer forecasts horizon are produced, the Wasserstein model shows a quite stable prediction error for the one, two and three-step ahead forecasts.

## Conclusion

This article introduce a novel approach to perform joint density forecast estimation. The methodology is grounded on OT and Wasserstein barycenters. The technique we propose is designed to learn the underlying joint distribution dynamic among a set of variables through Wasserstein barycentric coordinates. Accordingly, the learned generative mechanism is employed to predict the future configuration by exploiting the estimated barycentric coordinates coefficients and the contemporaneous values contained in the time series. This methodology, besides providing point estimates, yields one-step and multi-step head predictions of the joint probability. We apply this methodology to study the relationships between the market performance of the renewable energy sector and that of the fossil fuel energy. We focus on the behavior of the European Renewable Index and the MSCI Europe Energy. We find that the predicted joint distribution suggests market efficiency on the long-run, while on the short-run we observe some departures during crisis phases, in which the joint distribution moves towards negative returns. The empirical experiments that we design reveal the capability of the Wasserstein methodology to describe the joint dynamic of the indices and to outperform benchmark linear models, generally adopted for time series prediction. Moreover, as future research we will exploit the Wasserstein regression to construct counterfactual predictive densities by alterating the cost structure. Comparing the (counterfactual) of the conditional density after a perturbation of the cost matrix in the one-period-ahead predictive density allows us to build the density impulse response function, tracking how the entire joint distribution responds dynamically to an initial shock. This impulse can be used in a variety of settings, from evaluating the potential policy effects to constructing dynamically consistent stress testing scenarios.
